# Characterization of a Laterally Oscillating Microresonator Operating in the Nonlinear Region

**DOI:** 10.3390/mi7080132

**Published:** 2016-08-02

**Authors:** Aditya Ramanan, Yu Xuan Teoh, Wei Ma, Wenjing Ye

**Affiliations:** Department of Mechanical and Aerospace Engineering, Hong Kong University of Science and Technology, 1 Clear Water Bay Road, Kowloon, Hong Kong, China; aramanan@connect.ust.hk (A.R.); yxteoh@connect.ust.hk (Y.X.T.); vivian.w.ma@hotmail.com (W.M.)

**Keywords:** microresonators, nonlinear modelling, air damping, pressure sensing

## Abstract

Microresonators are popular structures used in a variety of applications. They generally operate in the linear region where the vibration amplitude is limited, thereby limiting the signal-to-noise ratio. The nonlinear vibration region, where amplitudes and, consequently, the signal-to-noise ratio are relatively large, is generally avoided owing to instabilities and complexities in analysing the vibrations. In this work, a nonlinear dynamic model with a damping constant obtained from Monte Carlo simulation was derived to describe the vibration responses of microresonators operating in the nonlinear region. A laterally oscillating comb-drive driven resonator was designed, fabricated and characterized at various pressures and driving signals to validate the model. A simple method to extract the quality factor of the resonator in the nonlinear region was also proposed. The measured quality factors were compared with those obtained from the nonlinear model and a good agreement was obtained.

## 1. Introduction

Micro-Electro Mechanical Systems (MEMS) have been a continually growing field thanks to the advanced microfabrication techniques, which facilitate the miniaturization of various devices. One of the major applications of MEMS technologies is for developing sensors utilized in a variety of fields [1]. Microresonators are a popular MEMS structure for sensing applications, particularly in mass, chemical, biological and motion sensing [2]. These devices are effectively mechanical structures that oscillate/vibrate at resonant frequencies. Their performance is often characterized by a parameter known as the quality factor (*Q* factor), which is defined as the ratio of energy input into the system to the energy lost from various damping sources such as air damping, anchor loss, thermoelastic damping, surface and intrinsic losses.

(1) $$Q=2\pi \frac{\text{Input Energy}}{\text{Energy Loss per Cycle}}$$

Typically, resonator based sensors are designed to function in the linear vibration region. The nonlinear region is often avoided because of instabilities and the complexity in analysing the vibration. However, to ensure a linear vibration region, the vibration amplitude needs to be limited to a small value, which leads to a small signal-to-noise ratio and consequently less accurate measurements. In addition, in some applications, such as energy harvesting, a large vibration amplitude is highly desirable and the nonlinearity in the microresonator can be exploited to improve the performance [3,4]. For these reasons, the possibility of using the nonlinear region to their advantage should be explored and some work has already been conducted along this direction [5]. 

To fully and accurately utilize the nonlinear operating range, a careful characterization of microresonators must be first conducted. One key task is the determination of the *Q* factor from the measured vibration amplitudes. In cases when the dominant damping factor is air damping, vibrations in resonators are governed by the wave equation, which, in the case of a linear vibration, simplifies to a second-order linear ordinary differential equation that can be easily solved to obtain the relationship between the amplitude and quality factor [6,7]. In the nonlinear region, the solution to the wave equation is complicated by the presence of the nonlinear terms [8], and there is no analytical expression that links the vibration amplitude and the quality factor, hence no easy way to extract the quality factor from the measured vibration amplitudes. In this work, a microresonator was designed and fabricated for the purpose of nonlinear characterization. A nonlinear model with a damping constant obtained from Monte Carlo simulations was derived to model the vibration responses of the resonator. A simple method based on the original definition of *Q*, Equation (1), was proposed and employed to extract the *Q* for the nonlinear resonator. This method was validated by comparing the experimental results with numerical simulations. 

The organization of the paper is listed as follows. Section 2 deals with the design and modelling of the resonator, which include a finite element method (FEM) structural analysis of the resonator and a Monte Carlo simulation of air damping. The fabrication process of our testing device will be discussed in Section 3 along with the experimental sensing scheme followed by the results, comparison and analysis in Section 4. 

## 2. Design and Modelling

**Figure 1:**
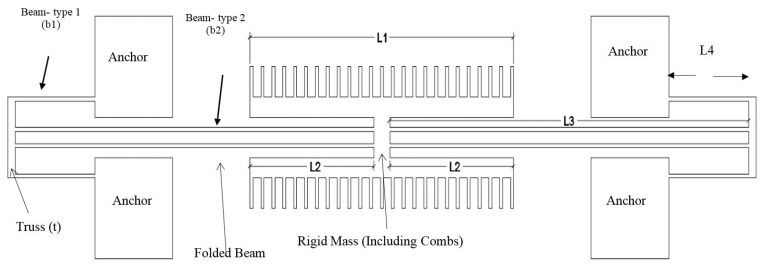
Top view of the microresonator structure.

The resonator is designed as a clamped-clamped structure with a comb-drive used to electrostatically drive and sense the resonator. The comb drive has a linear relationship between the electrostatic force applied and displacement and also facilitates large vibration amplitudes for which reason it was chosen. It is designed to excite the lateral vibration mode with a large amplitude without encountering instabilities. The plan view of the resonator is shown in Figure 1 and the finalized design dimensions are seen in Table 1. The suspended structure consists of two combs connected through a centre rigid mass and four folded beams anchored at one end of each beam as can be seen in the figure. The folded beam approach allows for softer structures to be fabricated, which vibrate with larger amplitudes given the same applied force. This in turn allows nonlinearities to be more easily observed. 

### 2.1. Lumped Model of Microresonator

The motion of the resonator is governed by the wave equation [7]. For the structure shown in Figure 1, a simple nonlinear mass spring damper system can be used to describe the vibration of the resonator. The general form of the equation is shown in Equation (2) [9].

(2) $$\begin{array}{l} m_{\mathrm{eff}}\ddot{x}+c\dot{x}+k_{\mathrm{eff}}x=F_{d}\;\\ k_{\mathrm{eff}}=k_{1}+k_{2}x+k_{3}x^{2} \end{array}$$

where $m_{\mathrm{eff}}$ is the effective mass of the system, c is the damping coefficient, $k_{1},\;k_{2}\;\text{and}\;k_{3}$ are the spring constants of the system, x is the vibration amplitude of the centre mass and $F_{d}$ is the external force applied to the system. In this case, $F_{d}$ refers to the electrostatic force applied to the resonator.

In the cases of small vibrations, the nonlinear spring force is much smaller than the linear spring force, and thus can be neglected. The equation is then simplified to a second order linear ordinary differential equation that can be easily solved analytically in the frequency domain to obtain the vibration amplitude, which relates with the quality factor as:

(3) $$\left|x\right|=\text{abs}\left(\frac{\left|F_{d}\right|}{k_{1}\left(1-\frac{\omega^{2}}{\omega_{0}^{2}}-\frac{j\omega }{\omega_{0}Q}\right)}\right)$$

where $\omega_{0}$ is the resonant frequency, $x=\left|x\right|e^{-j\omega t}$ and $F_{d}=\left|F_{d}\right|e^{-j\omega t}$.

**Figure 2:**
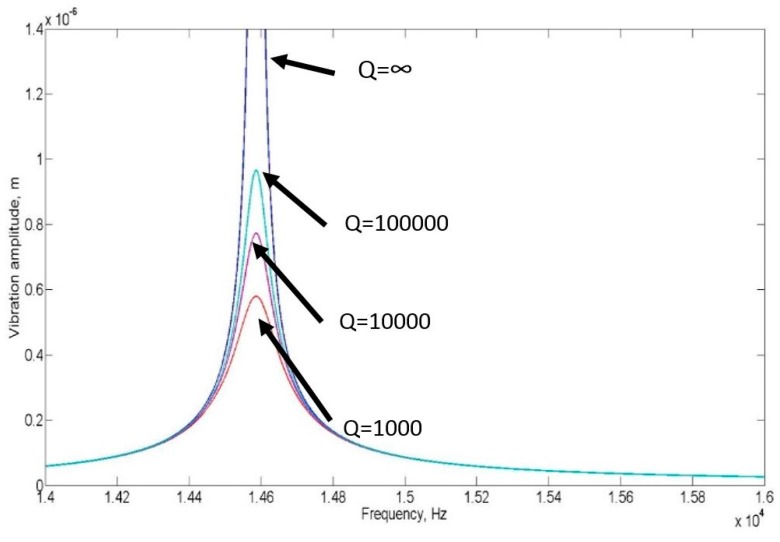
Vibration amplitude and Quality Factor against Frequency for a linear vibration, $k_{eff}=k_{1}$ .

Figure 2 illustrates several solutions of the linear form at various damping levels. As the amplitude increases, the nonlinear spring force increases and becomes comparable to the linear force, and hence the equation can no longer be simplified into a linear ordinary differential equation. 

### 2.2. Analytical Solution to the Duffing Equation

For the resonator structure shown in Figure 1, the quadratic nonlinear spring force can be shown to be much smaller than the cubic nonlinear force, and hence Equation (2) is simplified into the well-known Duffing equation. The analytical solution has been solved previously by Kaminski et al. [10]. In this paper, we obtain the solution through a similar method but it is more specific to our resonator. A variable $\delta_{f}$ is introduced to differentiate the motional displacement from any other displacement. In our case we assume the only displacement is motional in nature as we employ a differential driving scheme whereby the static force applied to the resonator from both electrodes is identical and cancels each other. Details will be explained in the next section. By substituting $\delta_{f}$ into Equation (2) and neglecting the second stiffness term ($k_{2}x$), we obtain Equation (4):

(4) $$m\ddot{\delta_{f}}+c\dot{\delta_{f}}+k_{1}\delta_{f}+k_{3}{\delta_{f}}^{3}=F_{d}$$

The non-dimentionalised form of Equation (4) is shown in Equation (5).

(5) $$\ddot{\tilde{\delta_{f}}}+\frac{1}{Q_{L}}\dot{\tilde{\delta_{f}}}+\tilde{\delta_{f}}+\epsilon_{f}{\tilde{\delta_{f}}}^{3}=\frac{\left|F_{d}\right|}{gm{\omega_{o}}^{2}}\left(e^{-i\omega^{\prime }t^{\prime }}\right)=f_{of}e^{-i\omega^{\prime }t^{\prime }}$$

with $\tilde{\delta_{f}}=\frac{\delta_{f}}{g}\;;\;t^{\prime }=\omega_{o}t\;;\;\omega_{o}=\sqrt{\frac{k_{1}}{m}};\;\omega^{\prime }=\frac{\omega }{\omega_{o}}\;;Q_{L}=\;\frac{m\omega_{o}}{c}\;;\;F_{d}=\left|F_{d}\right|e^{-i\omega^{\prime }t^{\prime }}\;;\;\epsilon_{f}=\frac{k_{3}g^{3}}{k_{1}};f_{of}=\frac{\left|F_{d}\right|}{gm\omega_{o}{}^{2}}$.

In Equation (5), *g* is the initial gap and t is the instantaneous time. The solution of $\tilde{\delta_{f}}$ can be assumed to take the form of

(6) $$\tilde{\delta_{f}}=A_{f}e^{-i\omega^{\prime }t^{\prime }}+{A_{f}}^{*}e^{i\omega^{\prime }t^{\prime }}$$

where $A_{f}$ denotes a complex number and ${A_{f}}^{*}$ is its conjugate. Substituting Equation (6) into Equation (5) and solving yields Equation (7).

(7) $$-{\omega^{\prime }}^{2}A_{f}+\frac{1}{Q}\left(-i\omega^{\prime }A_{f}\right)+A_{f}+3\epsilon_{f}{A_{f}}^{2}{A_{f}}^{*}=f_{of}$$

By rearranging Equation (7) and taking the square of the norm of both sides, the equation can be simplified into the cubic polynomial form, as seen in Equation (8).

(8) $$9{\epsilon_{f}}^{2}{E_{f}}^{3}+6\left(h\left(\omega^{\prime }\right)\right)\epsilon_{f}{E_{f}}^{2}+\left[{\left(h\left(\omega^{\prime }\right)\right)}^{2}+{\left(\frac{\omega^{\prime }}{Q_{L}}\right)}^{2}\right]E_{f}-{f_{of}}^{2}=0$$

where $E_{f}={\left|A_{f}\right|}^{2}$, ${\left|A_{f}\right|}^{2}=\;A_{f}{A_{f}}^{*}$ and $h\left(\omega^{\prime }\right)=1-{\omega^{\prime }}^{2}$.

The cubic polynomial can be analytically solved [11] but for ease MATLAB is used in our case to obtain the real roots of the polynomial. The roots give a solution for the $E_{f}$, which needs to be converted into the mechanical amplitude. Since $A_{f}\;\mathrm{and}\;{A_{f}}^{*}$ are complex numbers, they take the form of $A_{f}=a+ib\text{ and }{A_{f}}^{*}=a-ib$. Substituting these definitions into Equation (6) simplifies to:

(9) $$\tilde{\delta_{f}}=2a\cos\left(\omega^{\prime }t^{\prime }\right)+2b\sin\left(\omega^{\prime }t^{\prime }\right)=K_{f}\sin\left(\omega^{\prime }t^{\prime }+\emptyset_{f}\right)$$

where $\emptyset_{f}=\tan^{-1}\frac{a}{b}$ and $K_{f}=\sqrt{{\left(2a\right)}^{2}+{\left(2b\right)}^{2}}=2\left|A_{f}\right|=2\sqrt{E_{f}}$. By dimentionalising $\tilde{\delta_{f}}$ back we can obtain the real magnitude of the amplitude, $\left|\delta_{f}\right|$.

(10) $$\left|\delta_{f}\right|=\;\left|x\right|=\;g\left|\tilde{\delta_{f}}\right|=gK_{f}=\;2g\sqrt{E_{f}}$$

**Figure 3:**
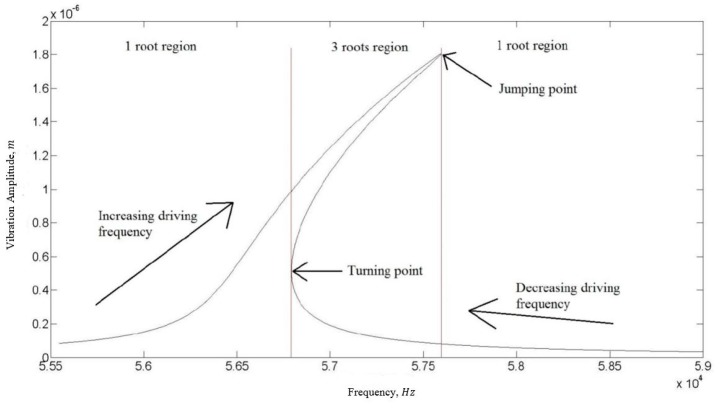
Amplitude response for a nonlinear resonator based on the solution to Equation (8).

**Figure 4:**
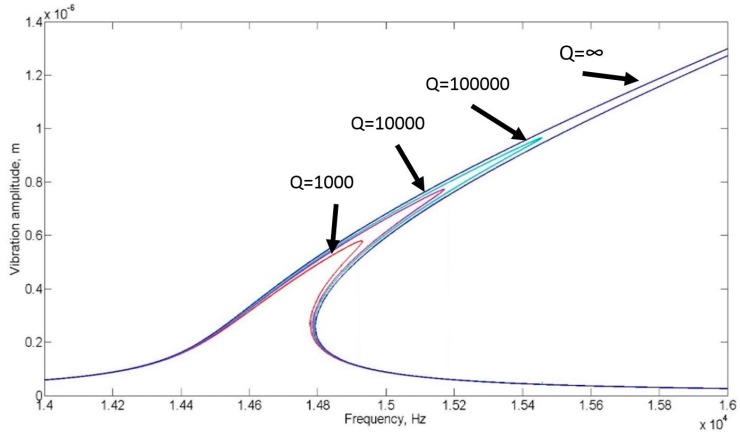
Vibration amplitude against Frequency for a nonlinear vibration at various damping levels indicated by the “linear” quality factor defined in Equation (5), $k_{eff}=k_{1}+k_{3}x^{2}$ .

Note that the final solution for the amplitude is a function of frequency and can be graphically expressed as seen in Figure 3. For frequencies below the turning point and above the jumping point, there is only one real solution to the equation. For frequencies between the turning point and the jumping point, the solution to Equation (8) yields three roots. In practice, only two solutions are realized, the maximum and the minimum depending on the direction of the sweep. The jumping point is dependent of the quality factor of the resonator. As the quality factor increases the maximum amplitude (jumping point) shifts higher and further to the right on the frequency spectrum as can be seen in Figure 4. When sweeping the frequency from low to high, the amplitude increases up until the jumping point is reached after which it drops to its minimum solution.

The final solution is a function of the effective mass, stiffness, force and damping, which will be defined in the following sub sections.

### 2.3. Effective Mass of System 

The effective mass is derived by employing energy principles and is similar to that derived by Tang et al. [12] for their resonator. The final result can be seen in the equation below.

(11) $$M_{\text{eff}}=M_{\text{RM}}+\frac{L_{4}{}^{6}}{{\left(L_{3}{}^{3}+L_{4}{}^{3}\right)}^{2}}M_{\text{truss}}+\frac{13}{35}\frac{L_{4}{}^{6}}{{\left(L_{3}{}^{3}+L_{4}{}^{3}\right)}^{2}}M_{b1}+\frac{\frac{13}{35}L_{4}{}^{6}+L_{3}{}^{3}L_{4}{}^{3}+L_{3}{}^{6}}{{\left(L_{3}{}^{3}+L_{4}{}^{3}\right)}^{2}}M_{b2}$$

The subscript “RM” refers to the rigid mass and “truss” refers to the two rectangular elements on either end of the resonator that connect the primary and secondary beams to each other as shown in Figure 1. The primary and secondary beams are represented by “*b*1” and “*b*2”, respectively. 

### 2.4. Static and Modal Analysis of the Microresonator

**Figure 5:**
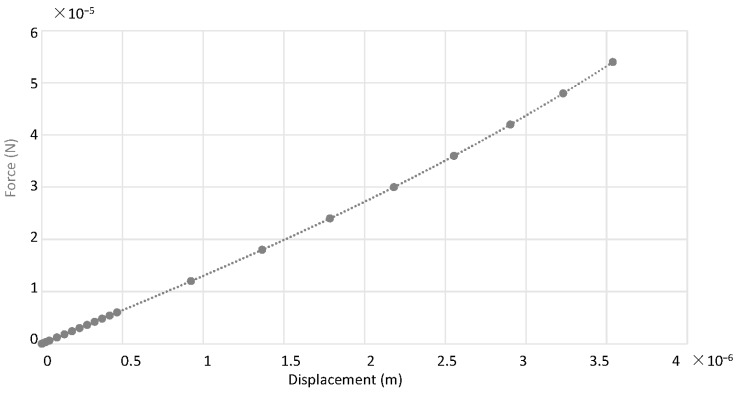
Plot of static spring force against displacement.

The linear and nonlinear stiffness constants, ($k_{1},\;k_{2}\;\text{and}\;k_{3}$) are obtained by performing a static finite element analysis (FEA) using CoventorWare (2014, Coventor Inc., Cary, NC, USA). The static analysis was performed by applying a force onto the centre of the resonator and the corresponding displacement was calculated. The static force to displacement curve is plotted from which the stiffness constants are determined. The curve can be seen in Figure 5.

Fitting the curve as a cubic polynomial, the equation for the static force is found to be Equation (12) where $F_{s}$ and $x_{s}$ are the static force and the displacement of the rigid centre mass, respectively

(12) $$F_{s}=(1.911\times 10^{11})x_{s}^{3}+(1.989\times 10^{4})x_{s}^{2}+\left(1.279\times 10^{1}\right)x_{s}+\left(9.701\times 10^{-10}\right)$$

Note that for a displacement $x_{s}$ between 0 and 4 µm, which is generally the range of the vibration amplitude in our case, the second and last terms are negligible compared to the first and third terms and hence can be neglected. Therefore, the effective stiffness for our resonator is

(13) $$k_{\text{eff}}=k_{1}+k_{3}x^{2}=\left(1.279\times 10^{1}\right)+\left(1.911\times 10^{11}\right)x^{2}$$

**Figure 6:**
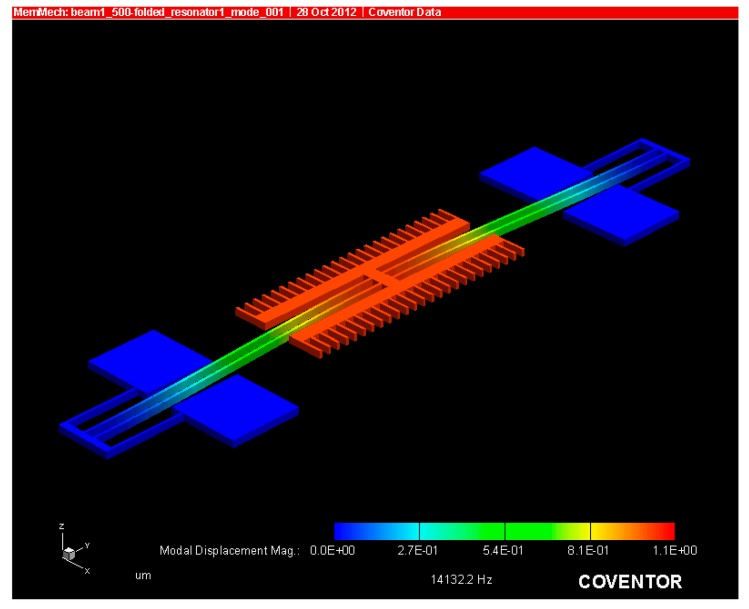
First resonant mode in the in-plane direction at a frequency of 14.1 kHz.

**Figure 7:**
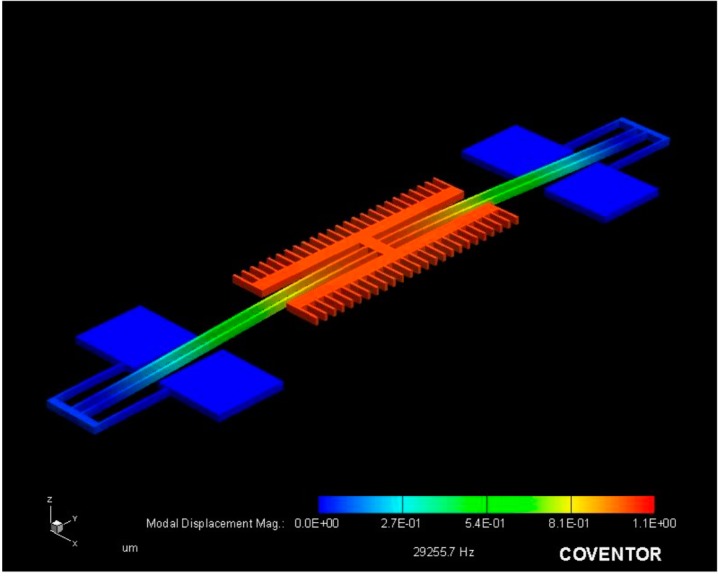
Second resonant mode in the out of plane direction at a frequency of 29.3 kHz.

The FEA analysis is also used to obtain the resonant modes of the device. The first two modes can be seen in the Figure 6 and Figure 7. Note that the resonant frequency of the second mode is nearly double that of the first mode, which was intentionally done to ensure no other modes are excited while driving the resonator in the in plane direction.

### 2.5. Electrostatic Force Acting on Resonator

**Figure 8:**
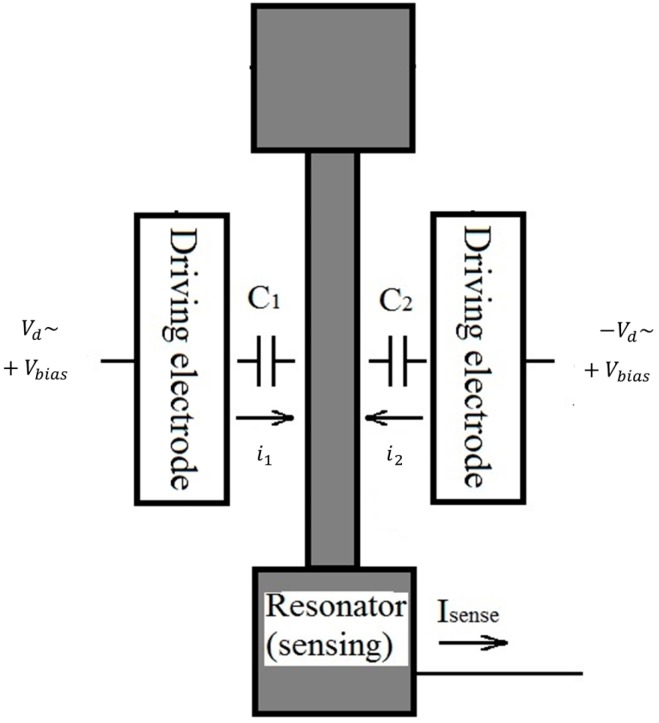
Simple schematic of a microresonator and the driving and sensing schemes.

The resonator is electrostatically driven via the application of a sinusoidal voltage and a bias voltage. For illustration purposes, the resonator device is divided into three parts, namely the left electrode, the right electrode, and the resonator beam as seen in Figure 8. The differential driving scheme, also shown schematically in Figure 8, is employed to drive the resonator. With the same bias voltage on both electrodes, the resonator vibrates around its neutral position with no change in stiffness. The electrostatic force acting on the resonator can be expressed as

(14) $$F_{d}=\frac{1}{2}\frac{dC_{1}}{dx}{\left(V_{\text{bias}}+V_{d}\right)}^{2}+\;\frac{1}{2}\frac{dC_{2}}{dx}{\left(V_{\text{bias}}-V_{d}\right)}^{2}$$

The capacitors formed between the resonator and electrodes are designated as $C_{1}$ for the left electrode and $C_{2}$ for the right electrode. By letting the positive x displacement of the beam be towards the right, $C_{1}$ and $C_{2}$ are defined as follows.

(15) $$C_{1}=\frac{N\varepsilon h\left(L_{0}-x\right)}{g};\;C_{2}=\frac{N\varepsilon h\left(L_{0}+x\right)}{g}$$

where N is the number of comb finger pairs, ε is the dielectric constant of air, h is the thickness of the structure, $L_{0}$ is the overlapping length between the resonator’s comb fingers and the electrode’s finger and g is the gap between the finger pairs. For our resonator, *N* is 50, *h* is 30 µm, $L_{0}$ is 20 µm and *g* is 4 µm. Substituting Equation (15) into Equation (14), the driving force can be determined

(16) $$F_{d}=2\frac{N\varepsilon h}{g}\left(V_{\text{bias}}V_{d}\right)=2\frac{N\varepsilon hV_{\text{bias}}}{g}\left|V_{d}\right|e^{-i\omega t}$$

### 2.6. Damping Analysis

When air molecules collide with a moving resonator, energy is transferred from the resonator to the air molecules leading to an energy loss known as air damping. It is a major loss mechanism for resonators oscillating in air or even in a low vacuum environment. In order to understand air damping, we must understand the behaviour and motion of the air molecules. A molecule’s motion is governed by its interaction with other air molecules (inter-molecular interactions) and its interaction with the surrounding walls (gas–wall interactions). The relative importance of these two interactions is characterized by a non-dimensional parameter known as the Knudsen number defined as the ratio of the molecule’s mean free path (λ) to the characteristic length of the system (d).

(17) $$Kn=\frac{\lambda }{d}$$

When the Knudsen number is above 10, the gas flow regime is known as the free molecular regime where gas–wall interactions are much more dominant than the inter-molecular ones and hence the latter interactions can be neglected. Practically speaking, the free molecular regime can be extended up to Kn>1. For our device, the pressure below which the free molecular assumption is valid is around 1700 Pa.

Air damping in our resonator can be separated into two parts, the first dealing with damping in an unbounded space and the second dealing with the comb fingers, which vibrate at a finite distance from each other.

#### 2.6.1. Air Damping of Structures Vibrating in an Unbounded Space

Except the comb fingers, the rest of the released structure can be considered vibrating in an unbounded space. Modelling of this case can be analytically determined and the method analysed by Martin et al. [13] is used.

The damping force, $F_{d}$ exerted by the air molecules, acting against the motion of the resonator structure can be defined by

(18) $$F_{d}=c\times u\left(t\right)$$

where “c” is the damping constant and u(t) is the velocity of the resonator in the x direction indicated in Figure 1 and Figure 6. In the free molecular region, the damping constant simplifies to Equation (19).

(19) $$c=\frac{P_{i}}{c_{s}}\left[A_{p}\left(\left(2-\sigma_{n}\right)\cdot \left(\frac{2}{\sqrt{\pi }}\right)+\sigma_{n}\sqrt{\pi }\right)+A_{s}\left(\frac{\sigma_{t}}{\sqrt{\pi }}\right)\right]$$

where $P_{i}$ is the ambient pressure, “$c_{s}$” is the speed of sound in the medium, $A_{p}$ is the area of the structure perpendicular to the direction of motion, $A_{s}$ is the area parallel to the direction of motion, and $\sigma_{n}$ and $\sigma_{t}$ are the normal and tangential accommodation coefficients, respectively. For our resonator, $A_{p}=\left[\left(L_{1}+2L_{2}+4L_{3}\right)\times h\right]$ where *h* is the depth of the structure and $L_{1}$, $L_{2}$ and $L_{3}$ are defined in Figure 1. $A_{s}$ is the summation of the bottom and top surface areas.

Under a perfectly diffuse boundary condition, that is, $\sigma_{n}=\sigma_{t}=1$, the force simplifies to

(20) $$F_{d,\text{Diffuse}}=\frac{P_{i}\cdot u\left(t\right)}{c_{s}}\left[A_{p}\left(\frac{2}{\sqrt{\pi }}+\sqrt{\pi }\right)+A_{s}\left(\frac{1}{\sqrt{\pi }}\right)\right]$$

Under a perfectly specular boundary condition, that is, $\sigma_{n}=\sigma_{t}=0$, the force simplifies to

(21) $$F_{d,\text{Specular}}=\frac{P_{i}\cdot u\left(t\right)}{c_{s}}\left[A_{p}\left(\frac{4}{\sqrt{\pi }}\right)\right]$$

The energy loss is obtained by calculating the work done by the damping force.

#### 2.6.2. Slide Film Damping between the Comb Fingers

The finite distance between the comb fingers introduces slide film damping for which the model derived in the previous subsection is no longer valid. Due to the rarefaction effect, Navier–Stokes equation is no longer accurate, and hence many well-developed methods and software packages cannot be applied. A numerical simulation method known as the Monte Carlo method is used to obtain the damping. The method used is the one developed by Hong and Ye [14]. It is valid in the free molecular regime and is more computationally efficient compared to Molecular Dynamic simulations. The simulation is split into discrete time intervals and during each interval the molecules are tracked and their energy exchanges with the resonator are recorded. The cumulative energy loss over the entire time period represents the total energy loss of the resonator.

The energy exchange between the resonator and the gas molecule is a function of the type of surface as well as the velocity of the resonator. The Maxwell gas–wall interaction model suggests that upon collision with a surface, a percentage of molecules are reflected specularly while the others are reflected diffusely. In the case of specular reflection, the energy and momentum of each individual molecule in the reference frame of the comb is conserved. Hence, the reflected velocity is derived from the conservation of momentum and energy and is seen by Equation (22) while the energy change can be determined from Equation (23) [15].

(22) $$v_{r}=2V_{\text{comb}}-v_{i}$$

(23) $$\Delta e_{\text{specular}}=\frac{1}{2}m\left(v_{r}^{2}-v_{i}^{2}\right)$$

m is the mass of a molecule, $v_{r}$ is the reflected velocity, $v_{i}$ is the incident velocity and $V_{\text{comb}}$ is the velocity of the moving comb which is the same as that of the resonator.

For a diffuse reflection, the reflected velocity follows the Maxwell distribution, characterized by the wall temperature, *T*, and the velocity of the resonator and is depicted by Equation (24) [16].

(24) $$v_{r}=\sqrt{\frac{k_{B}T}{m}}r_{g}+V_{\text{comb}}$$

where $k_{B}$ is the Stefan–Boltzmann constant and $r_{g}$ is a Gaussian distributed random number. 

The energy loss is calculated via the work done by the impact force upon collisions.

(25) $$\Delta e_{\text{diffuse}}=mV_{b}\left(v_{r}-v_{i}\right)$$

**Figure 9:**
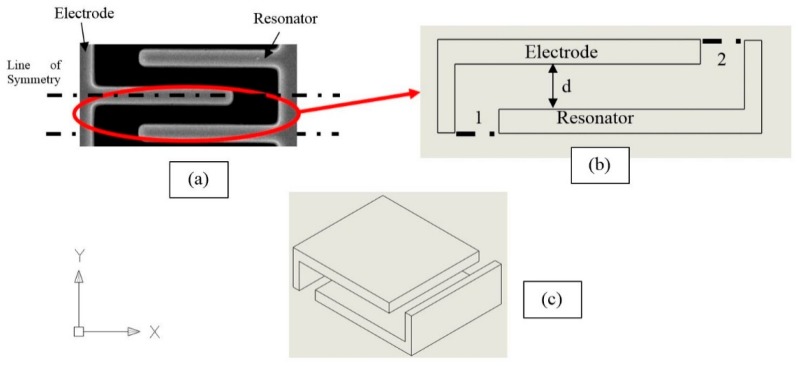
(**a**) 2D view of one set of comb fingers; the dash line is the symmetric line. (**b**) 2D illustration of the interaction domain/simulation domain. (**c**) 3D illustration of the simulation domain for Monte Carlo modelling.

The general algorithm is as follows. The system is initialized by defining the interaction domain, that is, the simulation domain, its physical properties and boundary conditions. To reduce the computational cost, a single comb was used to simulate the entire comb drive as seen in Figure 9. Since the comb shown in Figure 9a is perfectly symmetrical half way through as indicated by the dash line, the simulation domain can be further simplified to the interaction domain seen in Figure 9b,c by defining surfaces 1 and 2 in Figure 9b as a specular boundary. The resonator is assumed to vibrate with a sinusoidal amplitude in the lateral x direction.

At the beginning of each time interval, *N_u_* molecules enter into the simulation domain through the open surfaces on the top and the bottom of the combs. Assuming the ambient is in thermal equilibrium, the number of molecules entering the simulation domain is:

(26) $$N_{u}=\frac{1}{4}n\sqrt{k_{B}T}A$$

where “n” is the molecular number density of the gas, “*T*” is the temperature of the ambient gas and “*A*” is the area of the open surface through which molecules enter the interaction domain. 

Each molecule is then moved based on its velocity, and based on the location of the molecule and the comb boundary, it is determined if a collision with the comb takes place. If so, the molecular velocity is adjusted based on the properties of the comb and the new position is calculated based on this velocity and the remaining time. Once no collision is detected, the molecule’s position is updated solely based in its velocity.

The energy exchange during each collision is calculated based on Equation (23) or (25) and this energy is cumulated to measure the total energy loss per set of comb fingers over one oscillation period. This loss is multiplied by the total number of comb fingers to obtain the energy loss for the entire resonator due to the slide film damping between the combs.

**Figure 10:**
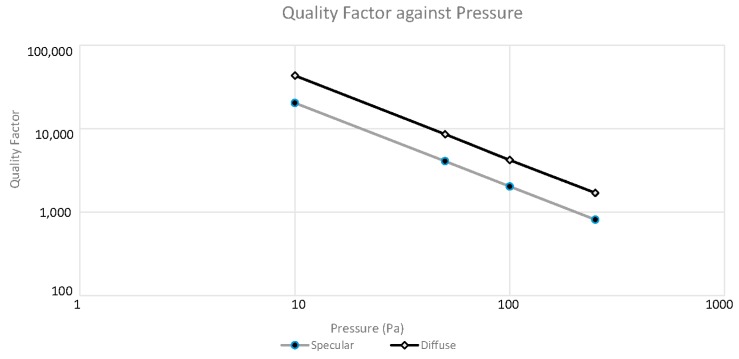
Quality factor of a resonator as a function of ambient pressure.

The total energy loss per cycle is the summation of the energy loss through slide film damping between the combs and the loss due to damping throughout the rest of the resonator vibrating in an unbounded space. The *Q* factor is then calculated according to Equation (1) where the input energy is the maximum kinetic energy of the system and all other losses are neglected. Figure 10 displays the simulated quality factor of a vibrating resonator as a function of the ambient pressure. Results corresponding to two extreme cases, pure specular and pure diffuse, are plotted. The theoretical slope of the curve should be one, which is indeed shown in both sets of results. 

### 2.7. The Quality Factor of a Nonlinear Resonator

As mentioned previously, the quality factor of a resonator with small vibration amplitudes can be obtained from the measured amplitude via Equation (3). This simple relationship however cannot be extended to the nonlinear case where the amplitude is large. In fact, it would be difficult, if not impossible, to obtain an analytical relationship between the amplitude and the quality factor. Hence in this work, the original definition of the quality factor, Equation (1), is used to extract the quality factor from the measured amplitude. In the case when air damping is the dominant damping factor, we realize that at any given frequency, the input energy of the system can be obtained by the non-damping amplitude, which can be calculated from Equation (8) by setting $Q_{L}=\infty$. Setting $Q_{L}$ to infinity is the same as setting the damping to zero. The measured energy is the energy remaining in the system and hence the difference between the input and measured energy represents the energy lost in one cycle. Equation (1) is then simplified to

(27) $$Q=2\pi \frac{E_{\text{in}}}{E_{\text{in}}-E_{\text{measured}}}=2\pi \frac{{A_{\text{in}}}^{2}}{{A_{\text{in}}}^{2}-{A_{\text{measured}}}^{2}}$$

where $A_{\text{in}}$ is the non-damping amplitude and $A_{\text{measured}}$ represents the measured amplitude with damping. This equation is used to determine the *Q* factor at a given frequency for the nonlinear vibrations. Note that $A_{\text{in}}$ is defined as the solution to Equation (8) assuming zero damping.

## 3. Fabrication and Experimental Setup

**Figure 11:**
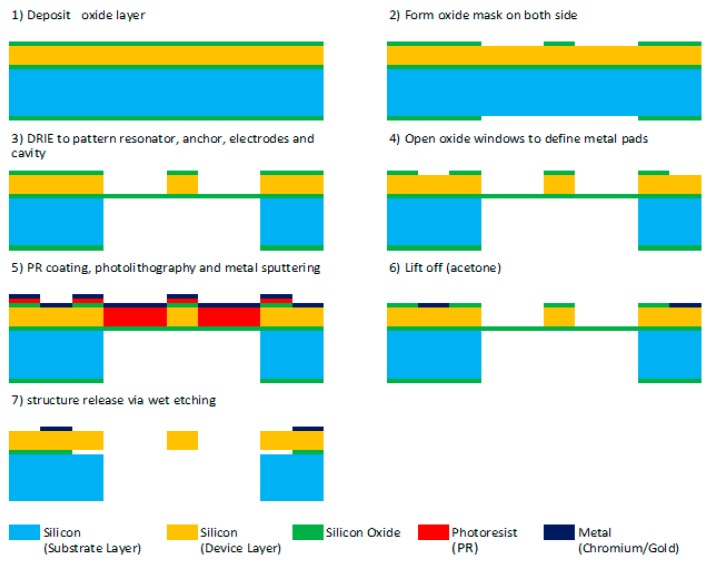
Fabrication process for the microresonator.

**Figure 12:**
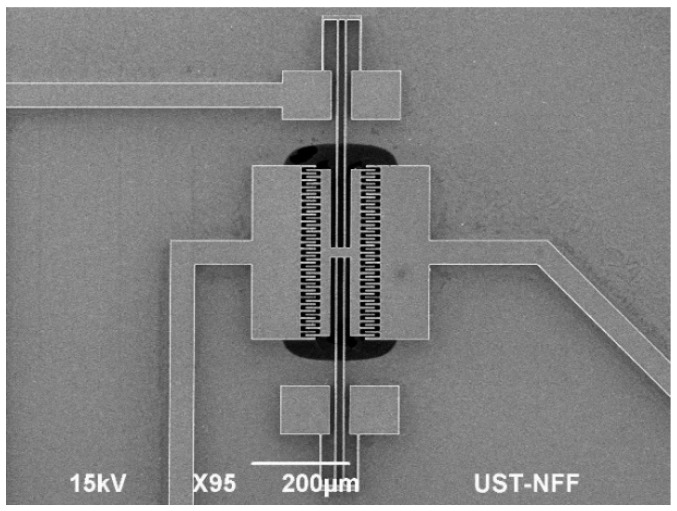
Scanning electron microscope (SEM) image of the microresonator. The dark region beneath the comb is a cavity.

The microresonator shown in Figure 1 was fabricated by employing various microfabrication techniques. Figure 11 illustrates the fabrication process. We began with a SOI (Silicon-On-Insulator) wafer on which we grew an oxide layer to behave as a hard mask for the etching processes. The second stage was to pattern the oxide mask with the aid of lithography machines. A mask was patterned on both sides of the wafer, of which the top had the resonator patterns on it and the bottom side had hole patterns to create a cavity beneath the resonator. A Dry Reactive Ion etch (DRIE) was performed to etch both sides as seen in Step 3 of the figure. The DRIE has the benefit of etching anisotropically which was essential in our case to etch through 30 µm. Photoresist (PR) was sprayed all over the surface after which openings were created on it and the oxide mask for the metal pads. As Step 5 illustrates, metal was then sputtered. After immersing in acetone for lift-off, the resonator was released using a buffered oxide etch (BOE) wet etch. The final released device is seen in Figure 12. 

**Figure 13:**
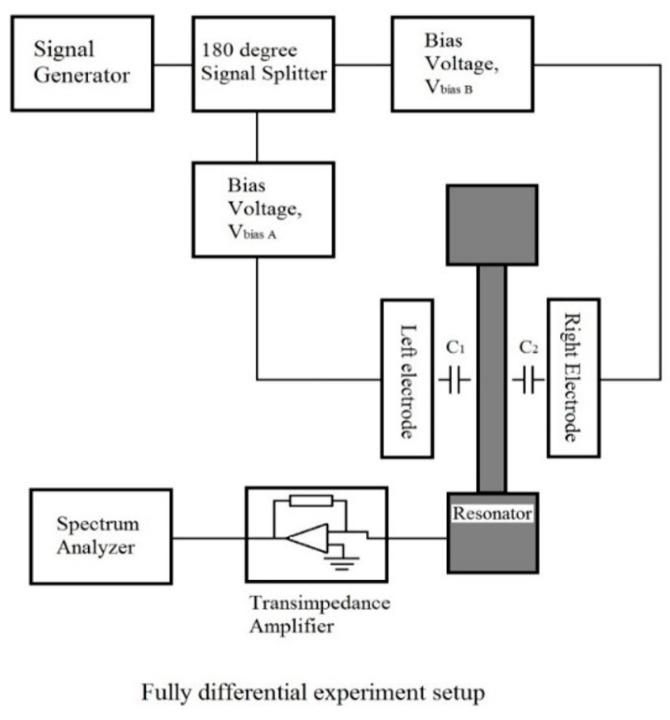
Block diagram of experiment with fully differential setup.

The performance of the fabricated resonator was characterized capacitively. The resonator was driven by the differential scheme explained in Section 2.5. A signal generator was used to generate a sinusoidal wave with an amplitude of $\left|V_{d}\right|$ which was split into two signals with a 180° phase difference between them. Both signals were added with a bias voltage, $V_{\text{bias}}$, before being input into the electrodes. The sensing current is then amplified and converted into a voltage by a transimpedance amplifier that can be detected in the spectrum analyser. Data are obtained for a range of frequencies around the resonant frequency. Figure 13 shows the block diagram of the experimental setup. 

The motion of the resonator produces a current from which the vibration amplitude is extracted. The measured current generally contains two parts: the motional component and the feedthrough current. The sensing current, $i_{\text{sense}}$, is shown in Equation (28), while $i_{1},\;i_{2}\;\mathrm{and}\;i_{sense}$ are defined in Figure 8. The terms with the vibration amplitude, x, represent the motional components

(28) $$i_{\text{sense}}=i_{1}+i_{2}\;\;i_{1}=\frac{d(C_{1}\left(V_{\text{bias}}+V_{d}\right))}{dt}=\left[C_{1}\frac{d\left(V_{\text{bias}}+V_{d}\right)}{dt}+\left(V_{\text{bias}}+V_{d}\right)\frac{dC_{1}}{dx}\frac{dx}{dt}\right]\;i_{2}=\frac{d(C_{2}\left(V_{\text{bias}}-V_{d}\right))}{dt}=\left[C_{1}\frac{d\left(V_{\text{bias}}-V_{d}\right)}{dt}+\left(V_{\text{bias}}-V_{d}\right)\frac{dC_{2}}{dx}\frac{dx}{dt}\right]$$

By substituting Equation (15) into Equation (28), the sensing current simplifies to Equation (29) assuming $x=\left|x\right|e^{-i\omega t}$ and $V_{d}=\left|V_{d}\right|e^{-i\omega t}$.

(29) $$i_{\text{sense}}=\frac{N\varepsilon h}{g}\left(\frac{dV_{d}}{dt}\left(2x\right)+\frac{dx}{dt}\left(2V_{d}\right)\right)=2\frac{N\varepsilon h}{g}\left|x\right|\left|V_{d}\right|\omega e^{-i2\omega t}$$

Note that the sensing current is at 2ω while the driving force shown in Equation (16) is at ω. Therefore, the feedthrough current and other parasitic currents that are at the driving frequency would not corrupt the motional current. The sensing current is converted into a voltage ($V_{\text{sense}})$ using a transimpedance amplifier and observed on the spectrum analyser. From the measured voltage, one can obtain the oscillation amplitude |x|.

## 4. Results and Discussion

The measurements were conducted inside a vacuum chamber at various pressure levels ranging from 0.5 to 400 Pa. This range of pressure is selected due to the potential application of the resonator for low-pressure sensing. The gas used inside the chamber was air. For the fully differential setup, the amplitude of the sinusoidal voltage applied to drive the resonator was 1.5 Vpp and the bias voltage was 2.25 V. The frequency sweep started from 7 kHz and stopped at 11.5 kHz. The sweeping time was set to 500 s at the signal generator. 

**Figure 14:**
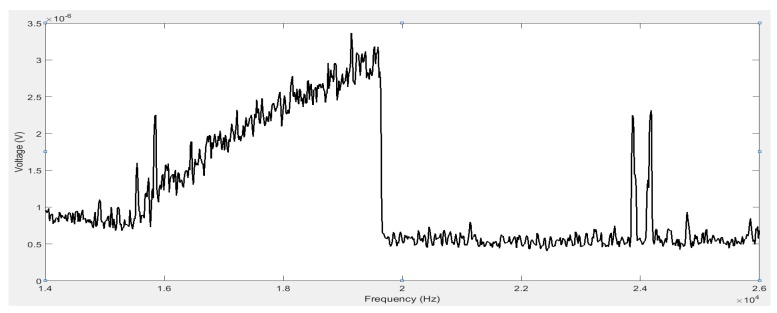
Voltage response in the frequency domain under driving conditions of (1.5 Vpp, 2.25 *V*_bias_) at 40 Pa.

**Figure 15:**
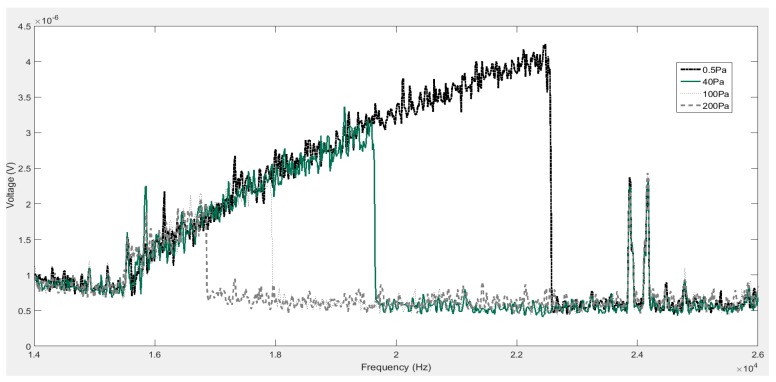
Frequency response of the microresonator at different pressures under driving conditions of 1.5 Vpp and 2.25 *V*_bias_.

As a representation, the voltage response taken at 40 Pa on the spectrum analyser is shown in Figure 14. Note that since the sensing current is at twice the driving frequency, the voltage response is also detected at 2ω. The profile of the curve is similar to that in Figure 3, demonstrating a typical Duffing nonlinearity. The resonator enters the resonant region at around 7.7 kHz, which corresponds to 15.4 kHz on the spectrum analyser. The maximum amplitude is reached at around 9.8 kHz, corresponding to 19.6 kHz on the spectrum analyser, beyond which the voltage drops to the minimum solution. The frequency and amplitude at which this drop occurs are related to the quality factor. This effect can be clearly observed in Figure 15, where four curves, corresponding to measurements at different pressures of P=0.5 , 40 , 100, and 200 Pa, are shown. As the ambient pressure decreases, the maximum amplitude increases due to the reduced damping.

**Figure 16:**
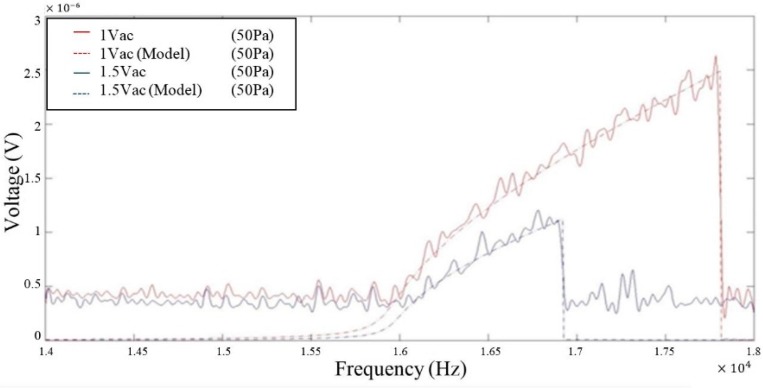
Matching of experimental data and Duffing solution under different driving voltages: 1 Vpp and 1.5 Vpp, at a pressure of 50 Pa.

In order to obtain the quality factor from the measured voltages, the linear stiffness ($k_{1}$), nonlinear stiffness ($k_{3}$) and effective mass are needed. While the theoretical values listed in Section 2.3 and Section 2.4 could be used, the real values are different due to variations in dimensions caused by the fabrication process and ideal boundary conditions assumed in the theoretical model. A test was conducted to calibrate these coefficients. The resonator was driven in an ambient pressure of 50 Pa and with two peak-to-peak driving voltages *V_d_* = 1 and 1.5 Vpp. The vibration amplitude responses, extracted from the measured voltage responses, were then matched with the solution to the Duffing equation by adjusting $k_{1}$, $k_{3}$ and damping constant *c*, and results are plotted in Figure 16. The theoretical value of the effective mass was employed as it was believed to be more accurate than the theoretical spring constants. 

In Figure 16, the solid lines are the experimental data while the dotted lines are the Duffing solutions. The values of $k_{1}$ and $k_{3}$ needed to achieve a good match in both cases are found to be 4.09 N/m and $1.4\times 10^{11}$ N/m^3^ respectively. The discrepancy between the two sets of results observed in the off resonance region is likely due to the noise from the spectrum analyser and the transimpedance amplifier which may have masked the signal. 

**Figure 17:**
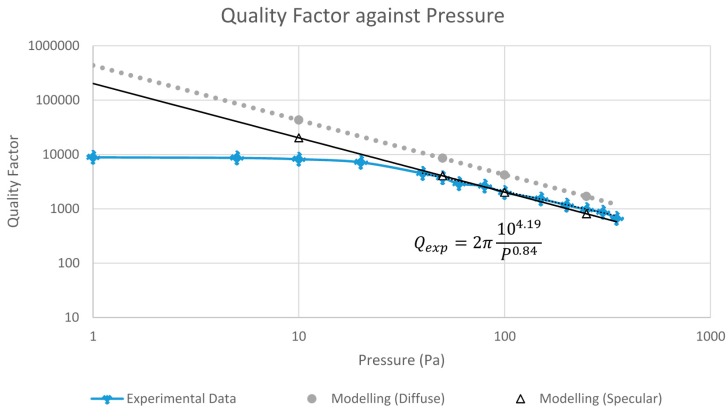
Quality factor plotted against pressure for our comb drive microresonator.

With the calibrated $k_{1}$ and $k_{3}$, the quality factors of the resonator at different ambient pressures were extracted using Equation (27). $A_{\text{measured}}$ was the oscillation amplitude |x| at the jumping point (defined in Figure 3) of the vibration response, that is, the maximum amplitude. The non-damping amplitude, $A_{\text{in}}$, was obtained from the solution of Equation (8) at the jumping frequency and by assuming the damping to be zero or in other words, $Q_{L}=0$. The calibrated $k_{1}$ and $k_{3}$ were used in the calculation. Figure 17 plots the measured quality factors together with the modelling results obtained in Section 2.6. Due to the lack of the information of the actual accommodation coefficients of the resonator, modelling results corresponding to two extreme cases, fully specular and diffuse reflections, are plotted. The real situation is expected to be in between the two cases. The experimental Q factor sensitivity to pressure is found to be 0.84, which is a 16% deviation from the theoretical value of one. The range where air damping dominates over the other loss mechanisms is for pressures above 20 Pa. Compared to the modelling results, overall the measured quality factors agree quite well with the modelling results with values been slightly on the low side. A main cause for this is due to the leakages in the detected signals. Our measured electronic signals were smaller than expected, most likely due to leakages in the current passing through the transimpedance amplifier fabricated in house. Additionally, the theoretical calculation of energy loss in Section 2 only assumes the presence of air damping and neglects other damping mechanisms that may also contribute to the difference in absolute values between the experimental and modelling results.

## 5. Conclusions

In this work, a microresonator vibrating in the nonlinear region was designed, fabricated and characterized, and a new way of extracting the quality factor from the measured data in the nonlinear region was proposed. A series of experiments were conducted under different driving voltages and ambient pressures to obtain the quality factors of the resonator, which are compared with modelling results from a nonlinear model. The good agreement between the two sets of the results indicates the feasibility of utilizing the nonlinear operating region, which would be beneficial in applications that require large vibration amplitudes. 

## Figures and Tables

**Table 1:** Summary of design dimensions.

L1	L2	L3	L4	Beam Width
340 µm	160 µm	463 µm	103 µm	4 µm
